# Effect of decrease of physical activity on depression and anxiety after the COVID-19 lockdown: A survey study

**DOI:** 10.3389/fpsyg.2022.961798

**Published:** 2022-11-17

**Authors:** Tanja Kajtna, Vojko Vučković

**Affiliations:** Faculty of Sport, University of Ljubljana, Ljubljana, Slovenia

**Keywords:** depression, anxiety, COVID-19 epidemic, sedentary behavior, physical activity

## Abstract

**Purpose:**

We focused on changes in the amount of physical activity (PA) and depression and anxiety symptoms in people, who were previously regularly physically active, as they were all members of fitness centers at time of lockdown because of COVID-19.

**Materials and methods:**

We sampled 150 fitness center members, tested individually in facilities of fitness centers. Depression and anxiety were measured with subscales of Personality Assessment Inventory (PAI) and PA was measured with global physical activity questionnaire (GPAQ).

**Results:**

We found that depression (*t* = −1.97; *p* < 0.05) and anxiety (*t* = −2.66; *p* < 0.05) was more present in female participants than male participants, single participants experienced more depression (*t* = 2.04; *p* < 0.05) than participants in relationship, unemployed participants experienced more depression (*F* = 3,24; *p* < 0.05) and anxiety (*F* = 5,32; *p* < 0.01) than employed participants and participants with lower levels of education experienced higher levels of affective depression (*F* = 3,42; *p* < 0.05) and physiological anxiety (*F* = 3,72; *p* < 0.05) than participants with higher levels of education. Finally, we found that mental health can be influenced by changes in amount of movement–both anxiety (*p* < 0.05) and depression (*p* < 0.05) (in whole and its specific dimensions) increased for male participants when there is less strenuous work-related activity, less walking, or cycling to work and when they would spend more time each day sitting. For female participants, affective depression (*t* = 3.78; *p* < 0.01) and anxiety (*t* = 3.23; *p* < 0.01) increased with increased sitting time. Ex-competitive athletes are particularly immune to anxiety (*t* = −2.18; *p* < 0.05) and depression (*t* = −2.09; *p* < 0.05).

**Discussion:**

As in some previous studies, our results show that because of lockdown, the most endangered groups for mood disorders are women, singles, unemployed and students, uneducated people and those, who had less PA, and more sitting time. Additionally, those who had some sport competitive history are less endangered for depression and anxiety.

**Conclusion:**

Isolation has great impact on mental health, the most effective solution to stress relief and anxiety is physical exercise, which was limited or non-existent in the time of pandemic. Ex-professional athletes are especially immune to anxiety and depression in events such as COVID-19 lockdown. For future studies we recommend focusing on likelihood of increased depression and anxiety levels in individuals, who were physically active before the isolation period.

## Introduction

Poor mental health and wellbeing is a major cause of disease burden globally, with depression considered one of leading contributors (Vigo et al., 2016). Regular exercise has been shown to improve health and reduce mortality (Haskell et al., 2007), caused by diseases such as cancer, type 1 and 2 diabetes and COPD (Malik et al., 2013; Sjøgaard et al., 2016).

However, it is often forgotten that physical exercise also has positive effects on the wellbeing and mental health of individuals (Thøgersen-Ntoumani et al., 2005; Lowenstein et al., 2015; Kandola et al., 2019; World Health Organization, 2020). Nevertheless, the majority of people still do not engage in sufficient physical activity (PA) (Hollasch et al., 2019). The situation created by the measures due to COVID-19 increases the inactivity and sedentary lifestyle of the population, which will only worsen the situation in future epidemics (Ammar et al., 2020; Hall et al., 2021).

Physical activity is any bodily movement produced by skeletal muscles that results in energy expenditure. This contains a large amount of activities like walking, cycling, gardening, sports, and much more (Buckworth, 2013). Many studies have shown the importance of physical activity in maintaining psychological health, or lack of physical activity with emotional problems (Kull, 2002; De Mello et al., 2013; Teychenne et al., 2020). Furthermore, Irandoust and Taheri (2018) suggested that physical exercise significantly improves quality of sleep components (quality of sleep, sleep latency, sleep duration, sleep efficiency, sleep disturbance, sleep medications, daytime dysfunction, and general quality of sleep). Dale et al. (2019) summarized through the analysis of 26 articles that physical activity is already crucial in reducing the symptoms of depression, anxiety, and poor self-esteem in children and adolescents. There are also positive effects of PA on the prevention of depression in older adults (Smaradottir et al., 2020). Even more, results from meta-analysis show us that PA and exercise has a moderate to large antidepressant effects also on clinical depressed population (Josefsson et al., 2014).

World Health Survey, including 47 countries (*n* = 237,964), has shown that low PA is connected with increased prevalence of anxiety (Stubbs et al., 2017). Also, meta-analytical evidence has shown that physical activity is associated with reduced risk of incident anxiety regardless of demographic factors (Schuch et al., 2019). Women (*n* = 5,180) that meet aerobic and resistance training recommendation are less likely to have depression/anxiety symptoms (Oftedal et al., 2019). Similar findings can be found in male population (*n* = 13,884), where it has been proven (Currier et al., 2020) that both increased duration and greater intensity of activity are related with further reduction in prevalence of depression. Therefore, promoting higher levels of physical activity is possibly a solution for improving men’s mental health.

Also, Marques et al. (2021) have proven that moderate physical activity and vigorous physical activity have positive effects on depression symptoms, even if performed only once a week, for both men and women. Similar conclusion was also in the study from Marques et al. (2020), who across a 4-year follow–up analyzed 32,392 European middle-aged to older adults, from 14 European countries. An interesting study was done by Pickett et al. (2012) who argued that leisure-time physical activity is a better remedy for depression than non-leisure time physical activity. The mentioned authors suggest that total energy expenditure is perhaps less relevant predictor of depression response than the psychosocial experiences of physical activity.

Furthermore, Fernandez-Montero et al. (2020) have proven that to prevent or reduce depression, duration of leisure-time physical activity is more important than the intensity. Several other factors are important in evaluating the effect of PA on preventing mood disorders. For instance, quality of the physical activity experience may be particularly essential to the mental health outcomes obtained (Lambert et al., 2018). Also, decreasing the visceral fat can be effective in improving sleep quality and can be beneficial to mental health (Irandoust and Taheri, 2018).

When a person is motivated by enjoyment or personal importance and choses to be active (i.e., autonomous motivation) it is more likely to have a positive effect, while PA undertaken due to guilt, pressure, or feeling forced (i.e., controlled motivation) can have negative effects on mental health (White et al., 2018). Furthermore, exposure to greenspace is associated with lower levels of depression, anxiety, and stress (Cohen-Cline et al., 2015). Consequently, PA outdoors is related with greater reductions in depression and anxiety, than PA indoors (Thompson Coon et al., 2011).

Another important factor in mood disorders prevention is sedentary behavior. Spending a lot of time sitting has been linked to more depressive symptoms (Rebar et al., 2014). Experimental evidence suggests that sedentary behavior can negatively affect mood detached of physical activity (Giurgiu et al., 2019). Meta-analytical evidence on 11 prospective studies (Zhai et al., 2015) has shown that the relative risk of developing depression/depressive symptoms was higher amongst those who engage in higher levels of sedentary behavior. van Uffelen et al. (2013) also proved that women who sat more than 7 hours/day and women who did no physical activity are more likely to have depressive symptoms than woman who sit less than 4 hours/day and did some physical activity. Abdul Rahim et al. (2022) highlighted the importance of improving PA and reducing sedentary behavior in improving student’s health and motivation to learn.

COVID-19 outbreaks have resulted in multiple cancelations in the sporting world (Dergaa et al., 2021). Lockdowns because of COVID-19 heavily limited people’s exercise possibilities and increased sitting time (Constandt et al., 2020; Wood et al., 2022). Although during the COVID-19 outbreak there were plenty of online information that home-based exercise was a safe way to perform physical exercise (Vancini et al., 2021), lots of people still did not engage in enough PA.

Lockdown had negative impact on mental health of adolescents (Barendse et al., 2021) and adults (Bueno-Notivol et al., 2021). Mental health during the COVID-19 pandemic has been shown to deteriorate, but there is a lack of research, which would look at changes in lifestyle form the perspective of physical activity during this pandemic. Specifically, there is little research on how changes in physical activity and increase of sedentary behavior is connected (or perhaps influences) mental health, specifically anxiety, and depression. One of the latest studies, made in Australia by Zhao et al. (2022) suggest that participants who met the physical activity guidelines had lower depression scores. None of the studies compared PA and mood disorders at the times of COVID-19 lockdown in people, who used to train competitive sport and people, who have never competed in any sport. Also, there were none of research about PA effect on depression and anxiety done in Slovenian geographic area.

The goal of this research is to look at the changes in the amount of changes in physical activity and depression and anxiety symptoms in people, who were previously regularly physically active, as they were all members of fitness centers.

## Materials and methods

### Participants

A total of 150 participants, all members of fitness centers, participated in the study, they were 33.32 years old in average (SD = 11.89 years). A total of 76 male participants (*M*_*age*_ = 33.30 let; SD = 12.04 years) and 74 female participants (*M*_*age*_ = 33.34 years; SD = 11.82 years) participated in the research. The differences in age we not statistically significant (*t* = −0.02; sig = 0.98).

Seven of our participants have completed grammar school, 53 high school, 32 higher school, 42 have university education, and 15 have a masters or a doctoral degree. Four unemployed participants participated in the research, 89 were employed, 19 were self–employed, 36 were students, and three were retired. A total of 61 participants were single and 89 participants of the research were in relationships.

A total of 84 participants in research had competitive careers in sport, in average of duration 8.89 years (SD = 4.44 years), while 64 participants had no competitive career. Male participants (*M*_*years*_ = 6.59 years; SD = 5.94 years) participated in competitive sport longer than female participants (*M*_*years*_ = 3.68 years; SD = 4.71 years) (*t* = 3.33; sig = 0.00).

### Instruments

#### Personality assessment inventory (PAI)–subscales depression and anxiety– Morey (2007, as cited in Gosar and Boben, 2009)

The personality assessment inventory (PAI) is a self-report questionary, which is designed of 344 questions. PAI evaluates various domains of personality and psychopathology among adults and was formed for use in professional and research environments. Items are completed on a four-point Likert-type scale.

The anxiety and depression scales of the PAI are each separated into three subscales: affective, cognitive, and physiological (Hill et al., 2013). The test–retest reliability of the individual PAI scales is reported as ranging from 0.85 to 0.94 for adults and 0.66 to 0.90 for students (Morey and Lowmaster, 2010). The cognitive anxiety subscale measures chances of harm and ruminative worry that may endanger an individual’s ability to concentrate. The affective anxiety subscale measures personal feelings of apprehension, tension, panic, nervousness, and difficulty in relaxing. The physiological anxiety subscale measures physical signs of anxiety. An overall anxiety scale represents all three expressions of anxiety.

The singular use of anxiety and depression subscales was allowed by the publisher of the Slovene version of PAI, Center za psihodiagnostièna sredstva–the permission was given specifically for this study.

#### Global physical activity questionnaire–World Health Organization (2021)

The amount of movement was measured in the metabolic equivalent (MET) unit, which was evaluated using the Global physical activity questionnaire (GPAQ) questionnaire on the general physical activity of the individual (World Health Organization, 2021). GPAQ is a standardized questionnaire suitable primarily for assessing moderate to high-intensity physical activity (Cleland et al., 2014). According to the World Health Organization (2021), the questionnaire contains three sets: activity during work, transport, and recreation. The amount of activity was labeled with MET, which expresses the intensity of physical activity and was used in the GPAQ questionnaire (World Health Organization, 2021). MET is defined as resting energy consumption (sitting) and for the average adult has value 1 kcal/kg/h (Ainsworth et al., 2011). When an individual is moderately intensely active, his calorie consumption is four times higher, and with high-intensity activity as much as eight times. Moderately intense exercise is therefore rated with 4 MET, high intensity with 8 MET. Data were refined and analyzed according to GPAQ guidelines for analysis of results (World Health Organization, 2021).

### Procedure

We tested participants individually in person during April 2021, they were tested individually in the facilities of fitness centers, they were approached upon coming to practice and asked for their willingness to participate. This research study was conducted in accordance with the Declaration of Helsinki (World Medical Association, 2013) and the Code of Ethics and Q4 Conduct of The British Psychological Society and all participants gave informed consent before taking part in the study. We processed the following data analyses with the statistical package IBM Statistical Package for Social Sciences (SPSS) 21.0: We computed differences between groups using *T*-test. We used multiple linear regression *via* ENTER method to predict anxiety and depression based on changes in amount of physical activity due to epidemic.

## Results

As shown in Table 1, affective, cognitive, and physiological anxiety is higher in female participants than the male participants, as well as is anxiety as a whole. The same applies for cognitive and physiological depression.

**TABLE 1:** Significant differences between male and female participants in changes in physical activity (PA) during epidemic and anxiety and depression.

	Male participants			Female participants			*t*	*sig*
	*N*	*M*	*SD*	*N*	*M*	*SD*		
Affective anxiety	75	5.73	3.76	71	8.37	4.87	−3.67	0.00
Cognitive anxiety	75	5.21	3.75	70	7.01	4.82	−2.50	0.01
Physiological anxiety	75	4.49	3.42	70	5.90	4.97	−1.97	0.05
Cognitive depression	76	3.36	2.46	71	4.51	4.01	−2.07	0.04
Physiological depression	74	5.73	3.80	71	7.14	4.79	−1.97	0.05
Anxiety	74	15.62	10.05	70	21.03	13.85	−2.66	0.01

*N*, number; *M*, mean; SD, standard deviation; *t*, value of statistical parameter t; sig, statistical significance of t.

Table 2 shows us the difference between previously competitively active and competitively non-active participants. Ex-competitors had more decrease in hours of strenuous work–related activity per week than non-competitors. Fewer affective anxiety symptoms were observed among ex-competitors and similar can be said for physiological depression, competitors had fewer symptoms than non-competitors. Competitors also had fewer depression symptoms than non-competitors.

**TABLE 2:** Significant differences between previously competitively active and competitively non-active participants in changes in physical activity (PA) during epidemic and anxiety and depression.

	Competitors			Non–competitors			*t*	*sig*
	*N*	*M*	*SD*	*N*	*M*	*SD*		
Decrease in hours of strenuous work–related activity per week	85	2.21	6.30	64	0.16	2.59	2.72	0.01
Affective anxiety	84	6.32	4.39	62	7.95	4.55	−2.18	0.03
Physiological depression	83	5.76	3.99	62	7.31	4.69	−2.14	0.03
Depression	83	13.34	9.43	61	16.90	11.00	−2.09	0.04

*N*, number; *M*, mean; SD, standard deviation; *t*, value of statistical parameter t; sig, statistical significance of t.

A significantly higher prevalence of affective depression symptoms was observed among single participants than among participants in relationships, they also demonstrated more depression than participants in relationships.

**FIGURE 1:**
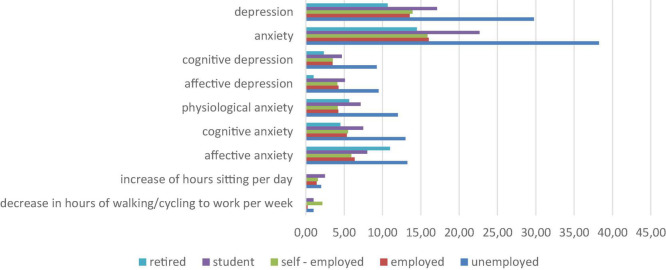
Significant differences in participants with different employment statuses in changes in physical activity (PA) during epidemic and anxiety and depression.

Figure 1 shows that the most anxiety and depression (in the entire scale as well as subscales) can be seen in unemployed participants of our study, the second group with highest presence of depression and anxiety (again, both in entirety and subscales) are students. This is also the group, where we can observe the highest increase in sedentary behavior. The greatest decrease in time spent walking or cycling to work was observed in self–employed participants.

**FIGURE 2:**
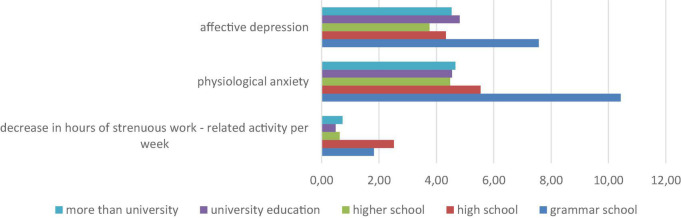
Significant differences in participants with different education in changes in physical activity (PA) during epidemic and anxiety and depression.

Figure 2 shows that people with lowest level of education (completed grammar school) experienced highest affective depression and physiological anxiety. In this dimension they were followed by participants with completed high school. In this group they experienced the largest decrease in hours of strenuous work-related activity. Table 3 shows that single participants are higher in affective depression and depression when compared to participants in relationships.

**TABLE 3:** Significant differences between single participants and those in relationships in changes in physical activity (PA) during epidemic and anxiety and depression.

	*N*	Single participants		*N*	In relationships		*t*	*sig*
		*M*	*SD*		*M*	*SD*		
Affective depression	59	5.57	4.37	87	3.81	3.80	2.51	0.01
Depression	59	16.92	10.77	85	13.41	9.66	2.04	0.04

*N*, number; *M*, mean; SD, standard deviation; *t*, value of statistical parameter t; sig, statistical significance of t.

Table 4 shows that changes in amount of movement have a strong impact on experiencing depression and anxiety–less strenuous work-related activity increases the amount of physiological anxiety and anxiety as a whole and an increase of cognitive depression. Less walking or cycling to work increases the amount of anxiety and affective depression, while increase in hours sitting per day increases appearance of physiological anxiety symptoms.

**TABLE 4:** Prediction of anxiety and depression based on changes in amount of physical activity (PA) due to epidemic for male participants.

	*Influenced by*	*B*	β	*T*	*p(t)*	*R*	*R* ^2^	*adj R* ^2^	*p(t)*
Physiological anxiety	Decrease in hours of strenuous work–related activity per week	−0.17	−0.30	−2.39	0.02	0.46	0.21	0.14	0.02
	Increase of hours sitting per day	0.41	0.25	2.01	0.05				
Affective depression	Decrease in hours of walking/cycling to work per week	0.33	0.25	1.99	0.05	0.45	0.20	0.13	0.02
Cognitive depression	Decrease in hours of strenuous recreational activity per week	0.13	0.27	2.02	0.05	0.42	0.17	0.10	0.05
Anxiety	Decrease in hours of strenuous work–related activity per week	−0.44	−0.26	−2.03	0.05	0.42	0.18	0.10	0.05
	Decrease in hours of walking/cycling to work per week	1.33	0.36	2.84	0.01				

*B*, unstandardized regression coefficient; β, standardized regression coefficient; *t*, t-statistic; p(t), *p*-value; *R*, coefficient of correlation; *R*^2^, coefficient of determination; adj *R*^2^, adjusted coefficient of determination.

Table 5 shows, that physiological anxiety and anxiety as well as the appearance of symptoms, described as affective depression, are all influenced by increase of hours sitting per day, thus meaning less movement of any intensity during the day.

**TABLE 5:** Prediction of anxiety and depression based on changes in amount of physical activity (PA) due to epidemic for female participants.

	*Influenced by*	*B*	β	*T*	*p(t)*	*R*	*R* ^2^	*adj R^2^*	*p(t)*
Physiological anxiety	Increase of hours sitting per day	0.98	0.39	3.19	0.00	0.48	0.23	0.15	0.01
Affective depression	Increase of hours sitting per day	1.00	0.46	3.78	0.00	0.48	0.23	0.15	0.01
Anxiety	Increase of hours sitting per day	2.84	0.40	3.23	0.00	0.47	0.22	0.14	0.02

*B*, unstandardized regression coefficient; β, standardized regression coefficient; *t*, t-statistic; p(t), *p*-value; *R*, coefficient of correlation; *R*^2^, coefficient of determination; adj *R*^2^, adjusted coefficient of determination.

## Discussion

Several studies have systematically shown the importance of regular physical exercise in the improvement of symptoms related to mood disorders (Thøgersen-Ntoumani et al., 2005; De Mello et al., 2013; Lowenstein et al., 2015; Kandola et al., 2019; Abdul Rahim et al., 2022). We wanted to research physical activity and sitting time influence on mood disorders, because it is known that the rates of positive screen for depression and anxiety during the lockdown in teens increased significantly to 64 and 50% in Greece (Giannopoulou et al., 2021) and 60% for depression and 53% for anxiety in China (Zhou et al., 2020).

To the best of our knowledge, this is the first study in which simultaneously physical activity and symptoms of depression and anxiety during COVID-19 lockdown have been appraised between previously competitively active and competitively non-active participants of the study.

Analyzing mood disorders during the lockdown, we found significant differences in anxiety and depression levels between male participants and female participants. We found that depression during lockdown is higher for female participants, as previously found in Turkey (Karaşar and Canlı, 2020), Mexico (Priego-Parra et al., 2020), and China (Xiao et al., 2020). Our study has also proven that anxiety symptoms would increase if being female, as previously found in Mexico (Priego-Parra et al., 2020), China (Hou et al., 2020; Xiao et al., 2020), and Iran (Hassannia et al., 2020). Our results show more significant difference between male and female on the field of anxiety than on depression. Some studies, on other hand, have found no statistical gender differences in depression and anxiety during COVID-19 lockdown (Wood et al., 2022). Research in the United Kingdom (Richardson et al., 2021) have proved increase in depression during lockdown for both male participants and female participants. Malaysian research showed us also that because of less PA than males, female students had more stress and were less motivated to study than male students (Abdul Rahim et al., 2022).

In results showing the difference between previously competitively active and competitively non-active participants of the study there was a significant difference showing us that non-competitors are less resilient to anxiety and depression. On one side, that may be because they are used to be in stress environment at the daily basis. And on other side, that may be because they are more fit.

Our results suggest that, in lockdown because of COVID-19, people who are single have a higher chance of exhibiting symptoms of affective depression and overall depression when compared to those who are in relationship. Some studies findings agree with our results (Lei et al., 2020), but others have not found depression to be significantly depending on relationship or marital status (Karaşar and Canlı, 2020).

Furthermore, our results agree with other studies that the severity of depression symptoms during COVID-19 lockdown would increase if a person is unemployed (Hou et al., 2020) or student (Karaşar and Canlı, 2020; Lei et al., 2020; Skapinakis et al., 2020; Rehman et al., 2021).

We confirmed the results from some other authors (Karaşar and Canlı, 2020; Lei et al., 2020), that lower education level is significantly associated with higher anxiety and depression during epidemic lockdown.

In the years before lockdown, several studies have systematically shown the importance of regular physical activity on mood disorders. De Mello et al. (2013) showed us that, when controlling all factors [gender, age range, socioeconomic class, education, and body mass index (BMI)], one of the most important predictors for the presence of depressive symptoms is the absence of regular physical activity. People are not physically active are 1.4 times more likely to exhibit symptoms of depression when compared with those who practice physical activity on a regular basis (at least three times per week). Also other author’s results show us the beneficial impact of PA in preventing anxiety (Hiles et al., 2017; Schuch et al., 2019; Marques et al., 2020) and depressive (Hiles et al., 2017; Marques et al., 2020; Smaradottir et al., 2020) symptoms. Furthermore, it is important to consider that there may be direct relationship between visceral obesity and mental health (Irandoust and Taheri, 2018).

Our results show that for male participants, physiological anxiety is negatively influenced by decrease in hours of strenuous work–related activity per week. Rebar et al. (2014) showed that people who spent more time sitting overall had more severe depression and anxiety symptoms than people who sat less. Our results confirmed that male participants who sit more during lockdown had bigger score on physiological anxiety. Also, evidence from France and Switzerland show us that increase of sedentary time during COVID-19 lockdown was associated with lower mental health (Cheval et al., 2021). Also Abdul Rahim et al. (2022) highlighted the critical role of PA in improving motivation or reducing stress through maintaining PA during the situation of lockdown, especially for females.

For male participants, decrease in hours of walking/cycling to work per week can cause affective depression and cognitive depression, which is directly positively influenced by decrease in hours of strenuous recreational activity per week. Anxiety symptoms are increasing if male participants decrease exercise in hours of walking/cycling to work per week. Therefore, our results for male population confirmed previous author results, significant impact of PA during the pandemic on depression (Currier et al., 2020; Wood et al., 2022) and anxiety (Wood et al., 2022).

Some authors have also found connection between PA and female participants depression (Fernandez-Montero et al., 2020), psychological wellbeing (Maugeri et al., 2020), or motivation to learn (Abdul Rahim et al., 2022) but we could not find significant impact of PA on mood disorders for women. But our results show that the increased sitting time because of COVID-19 lockdown significantly influenced physiological anxiety, affective depression, and anxiety at female participants. Authors from before lockdown had already proved that people who spent more time sitting overall had more severe depression and anxiety symptoms than people who sat less (Rebar et al., 2014). Similar studies were made also in lockdown (Romero-Blanco et al., 2020), and they proved connection between sitting time, depression, and anxiety.

## Limitations

The findings of this study have certain theoretical value and practical guidance but also have some limitations. First, this study is a cross-sectional study, the data were collected at once, and no effective follow-up study was conducted. Second, the sample size was 150 members of fitness centers, who were active before COVID-19, thus conclusions can be only generalized to some extent. Third, due to the cultural and sociological peculiarities of Slovenia, this study cannot be generalized worldwide. Fourth, as in all PA studies, participants were aware that they were asked about PA. They have been asked about both PA levels before COVID-19 and about PA levels at the time of gathering information. And last, also external factors could influence depression and anxiety levels: family problems, friendship problems, dietary habits and changes of environment/school, socioeconomic status, which have not been controlled for.

## Conclusion

Our results suggest that in all future epidemics that may come, maintaining physical activity and decreasing sitting time is crucial in preventing mood disorders like depression and anxiety. If sport clubs are closed, people should go out and exercise more, because contact with nature also reduces the likelihood of suffering from symptoms of depression and anxiety (Pouso et al., 2021).

We found that one of positive influences of training and competing in sports in younger years is also better immunity toward affective anxiety, physiological depression, and depression. Therefore, physical activity and sports are crucial for our defense against all other epidemics that are yet to come.

## Data availability statement

The raw data supporting the conclusions of this article will be made available by the authors, without undue reservation.

## Ethics statement

The studies involving human participants were reviewed and approved by Center za psihodiagnostična sredstva. The patients/participants provided their written informed consent to participate in this study.

## Author contributions

VV: conceptualization, writing—original draft preparation, project administration, and visualization. TK: methodology, software, formal analysis, supervision, and validation. VV and TK: investigation, resources, and writing—review and editing. Both authors have read and agreed to the published version of the manuscript.
